# Subjective and objective results 1 year after robotic sacrocolpopexy using a lightweight Y-mesh

**DOI:** 10.1007/s00192-013-2265-x

**Published:** 2013-11-22

**Authors:** Patrick J. Culligan, Emil Gurshumov, Christa Lewis, Jennifer L. Priestley, Jodie Komar, Nihar Shah, Charbel G. Salamon

**Affiliations:** 1 Atlantic Health System, Urogynecology and Pelvic Reconstructive Surgery, 435 South Street, Suite 370, Morristown, NJ 07960 USA; 2 Kennesaw State University, College of Math and Sciences, Kennesaw, GA USA

**Keywords:** Robotic sacrocolpopexy, Lightweight mesh, Prolapse, Transabdominal mesh, Pelvic organ prolapse

## Abstract

**Introduction and hypothesis:**

The objective of this study was to assess outcomes following robotic sacrocolpopexy using a lightweight polypropylene Y-mesh.

**Methods:**

During our study period, all patients who underwent robotic sacrocolpopexy were enrolled in this single-arm prospective trial. Endpoints included Pelvic Organ Prolapse Quantification (POP-Q) values; Pelvic Floor Distress Inventory, short form 20 (PFDI-20); Pelvic Floor Impact Questionnaire, short form 7 (PFIQ-7); Surgical Satisfaction scores; and the Sandvik Incontinence Severity Index. All surgeries were performed with a pre-configured monofilament type 1 polypropylene Y-mesh (Alyte©, C.R. Bard, Covington, GA, USA). Cure rates at 12 months were calculated using two separate definitions: (1) “clinical cure”: no POP-Q points > 0, point C ≤ −5, no prolapse symptoms on the PFDI-20, and no reoperations for prolapse and (2) “objective anatomic cure”: POP-Q stage 0 or 1, point C of ≤ −5, and no reoperations for prolapse.

**Results:**

A total of 150 patients underwent robotic sacrocolpopexy and 143 (95 %) were available for 12-month follow-up. Mean age was 58.6 ± 9.8 and mean body mass index was 26.3 ± 4.5. Mean operative time and blood loss were 148 ± 27.6 min (range 75–250 min) and 51.2 ± 32, respectively. There were no mesh erosions or exposures, and mesh edges were not palpable in any patient. At 12 months the clinical cure rate was 95 %, and the objective anatomic cure rate was 84 %. The PFDI-20 mean score improved from 98 at baseline to 17 at 12 months (*p* < 0.0001); PFIQ-7 scores improved from 59 to 6.5 (*p* < 0.0001).

**Conclusions:**

Robotic sacrocolpopexy using this lightweight polypropylene Y-mesh offers excellent subjective and objective results at 1 year.

## Introduction

Over the past 50 years, the sacrocolpopexy procedure has gone through quite an evolution. Originally an open abdominal procedure designed primarily for the correction of recurrent vaginal vault prolapse, the sacrocolpopexy is now often performed via the laparoscopic approach—with or without robotic assistance—for virtually any variety of pelvic organ prolapse (POP) whether or not the patient has a uterus [1]. It’s not only the route and scope of the procedure that have evolved—the graft materials used for the procedure have changed over time as well. A 2004 comprehensive review of sacrocolpopexy by Nygaard et al. essentially established the procedure as the de facto “gold standard” for POP [2]. In that report, over 80 % of the surgical cases referenced had been performed with one relatively heavy polypropylene mesh (Marlex, C.R. Bard, Covington, GA, USA). That particular mesh product has a density of 95 g/m^2^, which may partially explain the significant rates of mesh-related complications such as erosion (3.4 %), pain (2.7 %), and dyspareunia (8.7 %) contained in Nygaard et al.’s review. Recently, in an attempt to minimize mesh-related complications, medical device companies have developed much lighter-weight polypropylene mesh products with densities of less than 20 g/m^2^. All of these products received US Food and Drug Administration (FDA) approval for sacrocolpopexy via the 510(k) process, which means that no clinical research was required prior to release of these new lightweight meshes. While these products should result in fewer mesh-related complications, they beg the question “how light is too light?” In other words, will there be efficacy trade-offs when these lightweight mesh products are used for sacrocolpopexy? Obviously, each new lightweight mesh product should be scrutinized via prospective clinical trials in order to properly answer these efficacy questions. To that end our objective was to prospectively evaluate objective and subjective results at least 1 year after robotic-assisted laparoscopic sacrocolpopexy using a new lightweight Y-shaped polypropylene mesh material.

## Materials and methods

This single-arm prospective study was approved by the Atlantic Health System Institutional Review Board (R10-06-005) and was listed on the site www.clinicaltrials.gov (#NCT01320644). The inclusion criteria included patients who had been through our standard informed consent process and chosen to undergo robotic-assisted laparoscopic sacrocolpopexy. Exclusion criteria included enrollment in any other research study or desire to undergo sacrocolpopexy using a different graft material. All patients who underwent robotic-assisted laparoscopic sacrocolpopexy during our study period were enrolled in this trial. Participants’ demographic information was recorded including age, body mass index (BMI), ethnicity, history of prior prolapse or incontinence surgery and/or prior hysterectomy, smoking, and menopausal status. Due to our desire to perform supracervical rather than total hysterectomies (and thus morcellate the specimens), we maintained a low threshold of suspicion for uterine pathology. Any history suggestive of postmenopausal bleeding prompted us to perform an endometrial biopsy.

All surgeries were performed at Morristown Medical Center or Overlook Medical Center, which are community-based tertiary care teaching hospitals in northern New Jersey. Perioperative data were collected including concomitant operations, operative time, estimated blood loss, length of hospital stay, hospital readmissions, blood transfusions, and conversions to laparotomy. Any intraoperative or postoperative adverse events were recorded as well. All surgeries were performed via our previously reported standardized techniques [1, 11]. For each operation, the surgical team consisted of one attending surgeon and one urogynecology fellow. Briefly, the polypropylene Y-shaped mesh (Alyte® Y-mesh graft, C.R. Bard, Covington, GA, USA) was tailored to each patient’s defect such that it could be fastened down the anterior vaginal wall to the area adjacent to the trigone and down the posterior vaginal wall to the perineum. The mesh was fastened to the vagina using interrupted polytetrafluoroethylene sutures (CV4 Gore-Tex suture on TH-26 needles, Gore Medical Products Division, Flagstaff, AZ, USA). The proximal graft attachments to the anterior longitudinal ligament were performed via zero-gauge polyester sutures (Ethibond on SH needles, Ethicon, Somerville, NJ, USA), and the mesh was covered with peritoneum using zero-gauge poliglecaprone sutures (Monocryl on CT-1 needles, Ethicon, Somerville, NJ, USA). Operative time was defined as the time from incision to closure of the trocar sites. Concomitant suburethral slings were offered to patients who demonstrated stress incontinence with reduction of their prolapse on urodynamic studies.

In an effort to send our patients home without Foley catheter drainage, we employed a nonconventional voiding trial protocol. All Foleys were removed early in the morning of postoperative day 1, and the nurses were instructed to call the surgeon to report each voided volume. After each void, the surgeon would decide whether the given patient could be discharged home or needed to void again. If another void was called for, the nurse would call that value to the surgeon who would again decide whether the patient could be discharged home. In each case, the surgeon considered the particular patient’s preoperative voiding function, the voided amount, and the time interval since catheter removal or since the last void. This process was repeated for each patient until the surgeon either discharged her without a Foley catheter (because he/she believed the patient would continue to void well) or sent her home with a Foley for subsequent voiding trial in the office. In an effort to simplify the postoperative nursing duties, no post-void residual volumes were collected during this process. For patients who did require indwelling catheter placement at discharge, we recorded their days to spontaneous voiding.

Our clinical research nurse collected all outcome measures at the preoperative, 6-month and 12-month postoperative time intervals. Objective anatomic measurement of POP was performed via the Pelvic Organ Prolapse Quantification (POP-Q) system [3]. Subjective assessment of pelvic floor symptoms, sexual function, urinary incontinence severity, and surgical satisfaction were recorded via the Pelvic Floor Distress Inventory, short form (PFDI-20), the Pelvic Floor Impact Questionnaire, short form (PFIQ-7), the Pelvic Organ Prolapse/Urinary Incontinence Sexual Questionnaire (PISQ-12), the Sandvik Severity Index, and the Surgical Satisfaction Questionnaire (SSQ-8) [4–7]. During each postoperative vaginal exam, our clinical research nurse attempted to feel the mesh through the vaginal epithelium, and her findings were recorded as a yes/no dichotomous variable depending on whether she could discern the mesh edges. She also looked for evidence of mesh exposure at each postoperative visit.

Our primary outcome measure was “clinical cure” at 1 year. To meet our definition of clinical cure, all of the following were required: (1) no reoperation for POP since the index surgery, (2) no symptoms of vaginal bulge as measured by the PFDI-20, (3) no POP-Q points > 0, (4) POP-Q point C ≤ −5, and (5) an answer of “satisfied” or “very satisfied” on the SSQ-8. Unless all five criteria were met, a given patient was deemed a “clinical failure.” We also recorded a strictly POP-Q-based definition of cure we called “objective anatomic cure.” To meet that definition of cure, a patient had to have POP-Q point C ≤ −5, overall POP-Q stage of 0 or 1, and no reoperation for POP.

As secondary endpoints we recorded scores for the PFDI-20, PFIQ-7, Sandvik Severity Index, de novo dyspareunia rates, and SSQ-8. We used question 5 of the 12-month PISQ-12 data to define de novo dyspareunia among the study participants who were sexually active both before and after surgery. Question 5 asks “Do you feel pain during sexual intercourse?” We defined dyspareunia as an answer of ≥ 2 (“sometimes”).

Statistical analyses were performed using SAS 9.2 (SAS, Cary, NC, USA). The primary outcome was analyzed using Wilcoxon signed rank test and paired *t* test. Additionally, chi-square and Fisher’s exact test were used with the alpha set to 0.05.

## Results

Between August 2010 and May 2011, 150 patients underwent robotic-assisted laparoscopic sacrocolpopexy at our center using a lightweight monofilament polypropylene Y-shaped mesh (Alyte® Y-mesh graft, C.R. Bard, Covington, GA, USA). All eligible patients were enrolled, and no patients were excluded by criteria. Of these, 143 patients (95.3 %) returned for objective and subjective outcome assessment at ≥ 12 months; the remaining 7 patients (4.7 %) were lost to follow-up after their first postoperative visit. Table 1 lists preoperative characteristics of our study group. All patients who presented with an intact uterus received a supracervical hysterectomy as the first step of their sacrocolpopexy. Final pathology reports on the morcellated specimens revealed no unanticipated uterine pathology. Concomitant suburethral slings were placed in 122 (81.3 %) patients. Concomitant perineorrhaphies were performed in 17 (11.3 %) patients. These perineorrhaphies involved plication of the transverse perineal and distal bulbocavernosus muscles only. In other words, they did not include any posterior colporrhaphy stitches. Besides these perineorrhaphies, no other vaginal POP repairs were performed. There were no visceral injuries, blood transfusions, conversions to laparotomy, or readmissions to the hospital, and all patients were discharged home on postoperative day 1. The mean blood loss was 51.2 ± 32 ml, and the mean operative time was 148 ± 27.6 min (range 75–250 min). Only 2 % (3/150) of patients required indwelling Foley catheter placement at discharge, and each of these patients voided spontaneously without surgical intervention within the first postoperative week.

**Table 1:** Preoperative characteristics of the study group (*n* = 150)

Characteristic	
Age (years)	58.6 ± 9.8
BMI	26.3 ± 4.5
Vaginal parity (median)	2.5 ± 1.1
POP-Q stage	2.7 ± 0.6
PFDI-20 score	97.9 ± 21.2
PFIQ-7 score	59.3 ± 19.3
PISQ-12 score	32.9 ± 8.7
Postmenopausal^a^	76 (114/150)
Current smoker^a^	4 (6/150)
Preoperative systemic HRT^a^	3 (5/150)
Prior hysterectomy^a^	22 (33/150)
Prior prolapse surgery^a^	11 (16/150)
Prior continence surgery^a^	9 (13/150)
Caucasian^a^	97 (145/150)
African American^a^	0.67 (1/150)
Asian^a^	3 (4/150)

Data presented as mean ± standard deviation unless otherwise indicated

*HRT* hormone replacement therapy

^a^Proportion, % (*n*/*n*)

The clinical cure rate was 95 % (136/143). Two patients developed recurrent symptomatic anterior wall POP beyond the hymen, and two had recurrent symptomatic posterior wall POP beyond the hymen. The remaining three clinical failures included one patient whose POP-Q point C came to -4 (although she had no POP symptoms and reported being very satisfied with her surgery) and two patients whose POP-Q values were all at stage 0 or 1 but they reported persistent POP symptoms on PFDI-20.

**Fig. 1:**
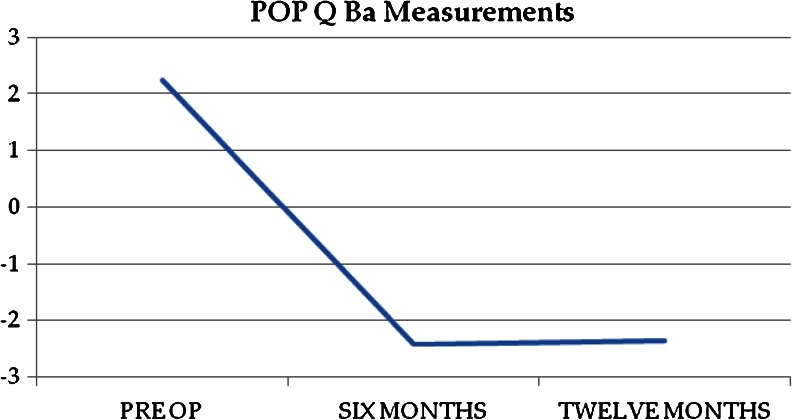
Point Ba over time

**Fig. 2:**
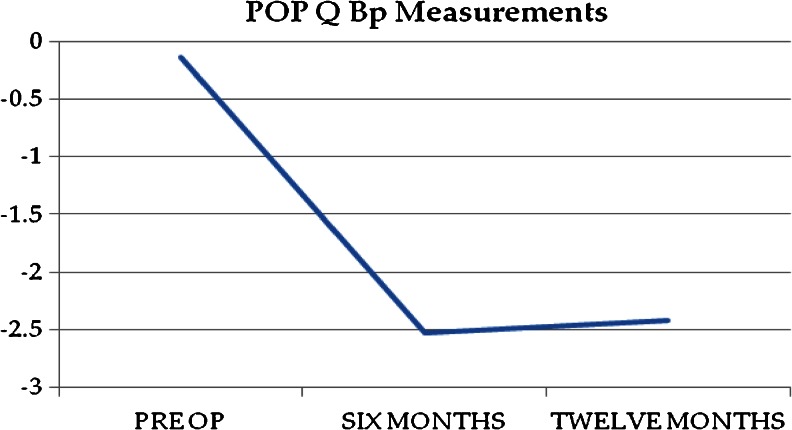
Point Bp over time

**Fig. 3:**
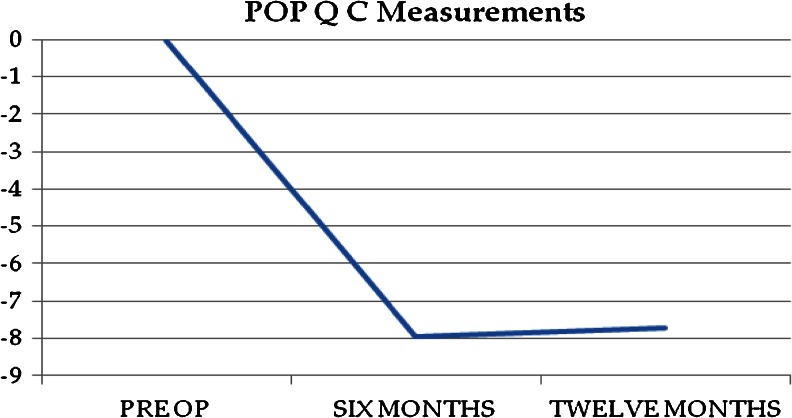
Point C over time

The objective anatomic cure rate was 84 % (120/143). Of the 23 objective anatomic failures, 15 were in the anterior compartment, 7 in the posterior compartment, and 1 at the apex. Figures 1, 2, and 3 show the mean POP-Q points Ba, Bp, and C over the study period. Table 2 includes pre- and postoperative comparisons for our secondary outcome measures.

**Table 2:** Pre- and postoperative data for the group of patients who completed ≥ 12 months follow-up (*n* = 143)

	Pre-op	12 months post-op	*p*
Point C	0.5 ± 4	−8 ± 2	<0.01
Point Ba	2.5 ± 4	−2.5 ± 2	<0.01
Point Bp	−0.7 ± 2	−2.5 ± 1	<0.01
TVL	9.4 ± 1	9.5 ± 1	1.0
GH	3.5 ± 2	2.6 ± 2	0.09
PB	2.5 ± 1	2.7 ± 1	0.7
PFDI-20	98 ± 19	17 ± 8	<0.01
PFIQ-7	58 ± 17	6.5 ± 5	<0.01
PISQ-12^a^	33 ± 9	42 ± 6	<0.01
SSI	2.3 ± 1	1.3 ± 1	<0.01

Data presented as mean ± standard deviation unless otherwise indicated

*TVL* total vaginal length, *GH* genital hiatus, *PB* perineal body, *PFDI-20* Pelvic Floor Distress Inventory, short form 20 [4], *PFIQ-7* Pelvic Floor Impact Questionnaire, short form 7 [4], *PISQ-12* Pelvic Organ Prolapse/Urinary Incontinence Sexual Questionnaire 12 [5], *SSI* Sandvik Severity Index (urinary incontinence) [8]

^a^Calculated for patients who reported pre- and postoperative sexual activity (*n* = 97)

On the SSQ-8, 96.6 % of patients reported that they were either very satisfied or satisfied and 96.4 % stated that they would recommend the operation to a friend. Sixty-four patients were sexually active without dyspareunia prior to surgery. Of these, three patients (4.7 %) reported de novo dyspareunia at 12 months. None of these patients had undergone a perineorrhaphy. Given the small number of patients with this outcome, statistical analyses looking for de novo dyspareunia risk factors were not feasible.

## Discussion

Our results indicated that use of the lightweight Alyte® Y-mesh for robotic-assisted laparoscopic sacrocolpopexy resulted in excellent anatomic and functional outcomes. These results were similar to historical results of the open abdominal and conventional laparoscopic approaches for the same operation [8–10] and were essentially identical to our recently published results for robotic sacrocolpopexies performed with other materials [1, 11]. Had we decided to count our seven patients who were lost to follow-up as failures, our clinical cure rate and objective anatomic cure rate would have been 91 and 80 %, respectively. The mean improvements in PFDI-20 and PFIQ-7 scores were well above the minimum clinically important difference (MCID) threshold reported by Barber et al. [4]. Furthermore, we achieved these cure rates in the face of no complications and minimal morbidity.

Our high rate of concomitant suburethral sling placement reflects our practice of offering this procedure to patients demonstrating stress incontinence during urodynamics while their prolapse is reduced. In our practice, the majority of such patients choose sling placement concomitant to their prolapse repair. All of these slings were the “bottom-up” retropubic type (Align®, C.R. Bard, Covington, GA, USA).

One of our most compelling findings was the lack of mesh-related complications during the first postoperative year. The absence of mesh exposure or erosion was probably a function of both the lightweight mesh used and the significant number of patients for which we performed a supracervical hysterectomy as the first surgical step. Furthermore, our clinical research nurse was not able to palpate the mesh through the vaginal epithelium in any patient. We attribute the absence of vaginal suture erosion or granulation tissue to our efforts to avoid full-thickness vaginal bites when securing the mesh, as we do not routinely ask patients to use postoperative vaginal estrogen.

It is also important to note that our simplified approach to establishing postoperative voiding status was well received by patients and nurses alike. By simply asking the nurses to report each voided volume for each patient we were able to eliminate the anxiety that often surrounded our previous voiding trial protocols (such as backfilling the bladder or checking post-void residuals). We were able to discharge 98 % of our patients home without indwelling catheters, and none of these patients required sling revisions or experienced voiding dysfunction thereafter.

The strengths of our study included the prospective design, the standardization of the surgical techniques, and the fact that all outcome measures were obtained by our clinical research nurse. Our lack of a randomization, lack of blinding (of either the clinical research nurse or the patients), and lack of a control group were our main weaknesses. Also, our choice of 12-month follow-up (rather than 3 or 5 years) could be thought of as a limitation despite the fact that the majority of POP recurrences after sacrocolpopexy happen during the first postoperative year [9]. Another important limitation of our study was the fact that our results may not be generalizable, because we used our specific standardized techniques and were through the robotic learning curve at the study outset. Nevertheless, as new products enter the marketplace via the FDA 510(k) process, studies like this one represent important steps in establishing the efficacy of these new materials. While the added expenses associated with use of the da Vinci robot may or may not prove to be justified, our 1-year outcomes are compelling at the very least. We intend to follow this group of patients over time to determine whether our early success will prove to be long lasting.
